# Extension of pole differential current based relaying for bipolar LCC HVDC lines

**DOI:** 10.1038/s41598-025-94842-0

**Published:** 2025-05-09

**Authors:** Ravi Shankar Tiwari, Jai Prakash Sharma, Om Hari Gupta, Marwan Ahmed Abdullah Sufyan

**Affiliations:** 1 Department of Electrical Engineering, GLA University, Mathura, Uttar Pradesh 281406 India; 2 Department of Electrical Engineering, NIT Jamshedpur, Jamshedpur, Jharkhand 831014 India; 3 College of Engineering and Information Technology, Aljanad University for Science and Technology, Taiz, Yemen

**Keywords:** Differential protection, Traveling wave, Fault location, Line-commutated converter (LCC), DC transmission line protection, Cross-country faults, Evolving faults, Electrical and electronic engineering, Energy grids and networks

## Abstract

Line commutated converter (LCC) based high-voltage direct current (HVDC) transmission is characterized by long-distance power transfer, a complex and harsh corridor environment, and the rapid fault evolution of DC lines. Since high fault currents can potentially cause unalterable destruction to power devices, fast and reliable line protection and isolation are essential to enhance the security and reliability of LCC-HVDC transmission systems. This paper combines an extended relaying using single- and double-ended measurements of the transient current at the boundary of positive and negative polarity DC lines to meet this obligation. During a fault, the DC line equivalent circuit is initially analysed with the time domain response of positive and negative polarity DC line (poles) currents at two ends to define local and global pole differential currents (PDCs). The criterion of relaying is then developed using PDCs for the bipolar and monopolar modes of operation of the LCC-HVDC transmission system. The proposed relaying method is validated through simulation, with results indicating that it can quickly (within 5.1 ms) and reliably detect internal faults and remain stable during external faults, accurately identify faulty poles without requiring data communication synchronization for bipolar operation, and demonstrate strong transition resistance. However, the monopolar operation uses global PDC to detect faults reliably within 15.6 ms. The proposed scheme is implemented on a 2000 MW, ± 500 kV, 900 km bipolar LCC-HVDC transmission system, simulated using PSCAD/EMTDC software. Additionally, the results are analyzed using MATLAB/Simulink.

## Introduction

### Motivation and incitement

The major energy resources in India are inversely distributed with the load centres. At the same time, high voltage direct current (HVDC) transmission is characterized by large capacity, long distance, and narrow transmission corridor^1^. Therefore, HVDC transmission plays a crucial role in enabling long-distance, high-capacity power transfer, expanding urban grids, interconnecting asynchronous systems, integrating isolated island networks, and delivering renewable energy. It holds a significant position in the modern power system, making its safe and stable operation essential for ensuring the power grid’s reliability^2,3^. Voltage source converter based-HVDC (VSC-HVDC) has the advantage of reliable electricity transmission from weak AC grids, free from the problem of commutation failure. However, VSC faces limited overload ability, low power transfer capacity and high switching losses. Consequently, the number of line-commutated converter-based-HVDC (LCC-HVDC) transmission corridors is still increasing in modern power grids. They are effective for large power transfers over long distances^4–7^. Nevertheless, faults in LCC-HVDC lines seriously threaten their safe operation. Thus, designing a suitable relaying scheme is essential for these systems.

### Literature review

Fault detection approaches in DC lines are generally classified as single-end and double-end relaying schemes^2,8–10^. The high susceptibility and controllability of the converter impose stringent demands on the operating speed of the relaying schemes. As a result, single-end relaying has emerged as a prominent research focus in recent years^11^. The single-end relaying schemes use one-end measurement to detect and classify faults. Moreover, single-end protection offers the benefit of rapid operation. It can serve as the primary protection method for an HVDC transmission system. Generally, single-end travelling waves are used as the primary protection for the LCC-HVDC lines. For example, ABB utilizes a voltage traveling wave’s magnitude and rate of change to establish the primary criterion for traveling wave protection, ensuring reliable differentiation between internal and external faults. In contrast, Siemens employs voltage variation and its rate of change to formulate the protection criterion^12^. Authors^13,14^ introduced a hybrid high-frequency transient traveling wave-based internal and external fault detection and classification in DC lines. The scheme is resilient to noise in traveling waves because it involves a stationary wavelet transform-based preprocessing unit to eliminate noises. However, these techniques necessitate an exceptionally high sampling rate of up to 50 kHz. Reference^15^ detects and identifies a fault in DC lines based on sequence components of current and voltage traveling waves. However, it can suffer the problem of low sensitivity for the far end and transition resistance faults. In^16^, a quick single-end protection based on transient voltage enhances the robustness against high fault resistance. However, the transient voltage or current-based techniques are vulnerable to noise and attenuate with propagation over the transmission network.

In^17^, a traveling wave-based directional pilot protection discriminates faults using the integral value of power and current at LCC and VSC ends, respectively, and the positive-to-negative pole currents ratio selects the faulty pole. However, the high sampling frequency of 1 MHz and reliable communication channels are the prime requirements for implementing these schemes. In^18^, an enhanced method was proposed to detect and classify faults based on peak clustering regression density for hybrid LCC-VSC HVDC lines. However, the protection can be affected due to computation complexity and high input data accuracy needed in the peak clustering density method. Reference^19^ proposes a selective and fast relaying based on instantaneous boundary impedance for three-terminal LCC-VSC HVDC lines. However, it can cause incorrect detection or misclassification because boundary impedance may change due to changes in load conditions, power flow, and converter operating modes. In^20,21^, the highly selective differential approach was proposed for detecting faults in LCC-HVDC lines. However, the scheme requires an extended delay to avoid incorrect trips due to distributed line capacitance. In^22^, an innovative differential protection has been suggested, focusing on compensating for discharging capacitive currents. Nevertheless, this approach is impractical for lengthy lines because it relies on the lumped parameter line model. In^23^, the time-domain calculation of differential currents is performed utilizing the distributed parameter line model. It used the Bergeron model of the DC line with the line parameters computed at zero Hertz. Consequently, the effectiveness of this approach profoundly relies on the accuracy of the line parameters during the transient period of a fault.

### Contributions and paper organization

To overcome the limitations of existing solutions, this paper proposes an LCC-HVDC line protection scheme based on the transient current of both healthy and faulty DC lines (i.e. positive and negative poles) and using the selective feature of both single-end and double-end protection philosophy. Initially, the current characteristics at both the rectifier and inverter ends of the DC line are examined. The findings reveal that the transient current signals experience significant attenuation due to transition resistance and the location of the fault.

Based on the transient current at the rectifier or inverter end of DC lines, i.e., at the positive and negative poles boundary, the local and global Pole differential currents (PDC) are defined, and their amplitude-time characteristics are analysed. Then, the local PDC polarity analysis is performed to select faulty poles.

The key contributions of this paper can be outlined as follows:

(i) Analysed the transient current–time characteristics from both ends of DC lines.
(ii) The theoretical analysis confirms the feasibility of the proposed method for a LCC-HVDC transmission system.
(iii) Analysis of the proposed scheme during both bipolar and monopolar operation modes.
(iv) Analyzed the Cross-country and evolving fault conditions for LCC-HVDC lines.
(v) Simple calculation, easy to implement practically and integrable with the existing HVDC relaying schemes.
(vi) No extensive training input–output data set is required as needed by machine learning (ML) techniques.
(vii) Low sampling rate requirement of 20 kHz compared to most traveling wave, wavelet and ML techniques.

To validate the effectiveness of the proposed approach, the paper presents a detailed study on system modeling, fault scenarios, and performance evaluation. The remaining part of this paper is organized as follows: Section II covers the configuration of the LCC-HVDC test system and the equivalent circuit of the HVDC link during internal fault conditions. Section III explains the relaying criterion of extension of the PDC scheme for bipolar and monopolar operation. Section IV presents the result and discussion, analysing the proposed scheme’s performance. Finally, Section V concludes the work, summarising the key findings and potential future directions.

## Configuration of HVDC link and equivalent circuit during fault

### Bipolar/Monopolar Configuration of LCC-HVDC Transmission

**Fig. 1:**
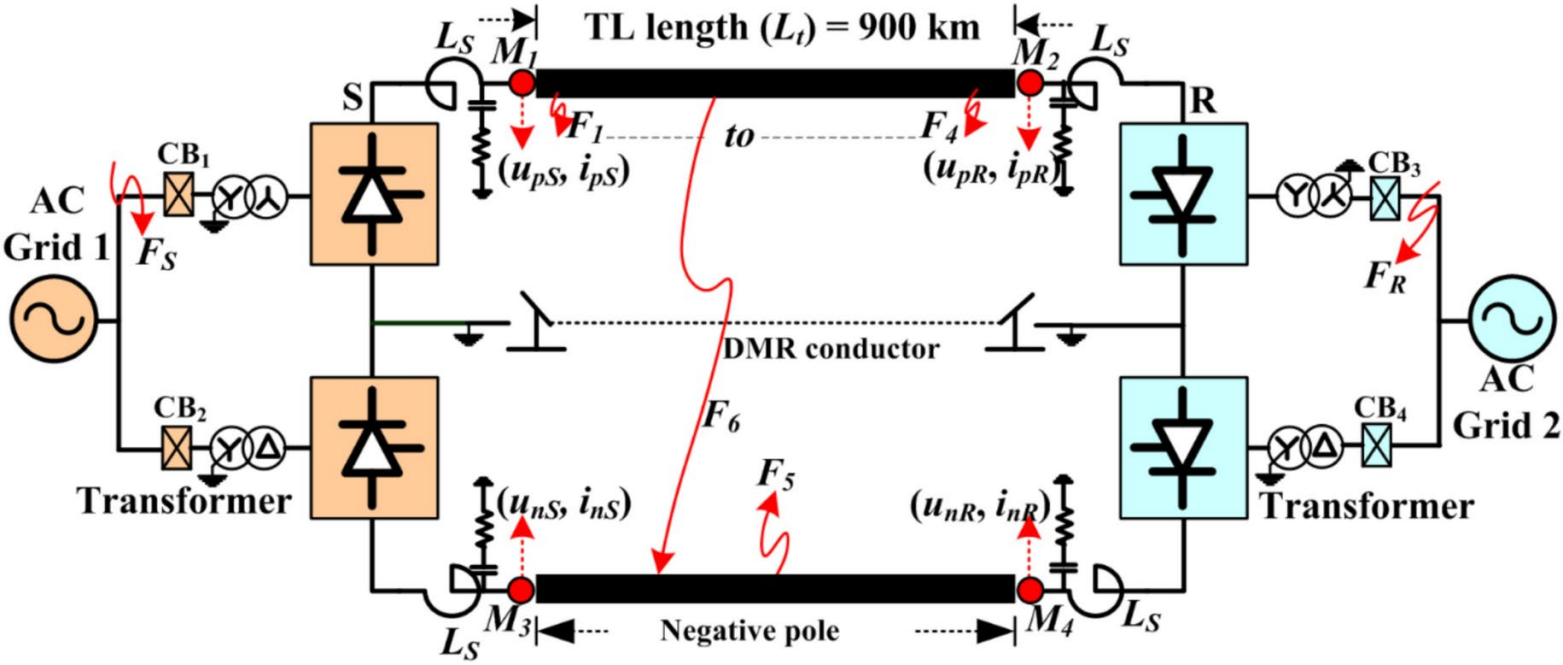
An emblematic one-line illustration of LCC-HVDC transmission system.

Figure 1 shows the bipolar LCC-HVDC transmission system used to perform the study proposed in this paper. The test system is rated at ± 500 kV, 1000 MW capacity, with a transmission length of 900 km^24^. The system follows the converter mid-point grounding with dedicated metallic return (DMR) provided for monopolar operating mode. The detailed parameters of the DC line, converter and power sources are illustrated in Tables A1 and A2 of the Appendix. The relying unit may installed at line ends *M*_*1*_, *M*_*2,*_ *M*_*3*_ or *M*_*4*_, governed by measured current at these locations. Multiple ground faults are simulated separately with varying fault locations, resistances, and operating modes to evaluate the performance of the proposed method. Subsequently, the results are analyzed in the MATLAB/Simulink environment.

### DC line equivalent during pole-to-ground faults (P-G-Fs)

**Fig. 2:**
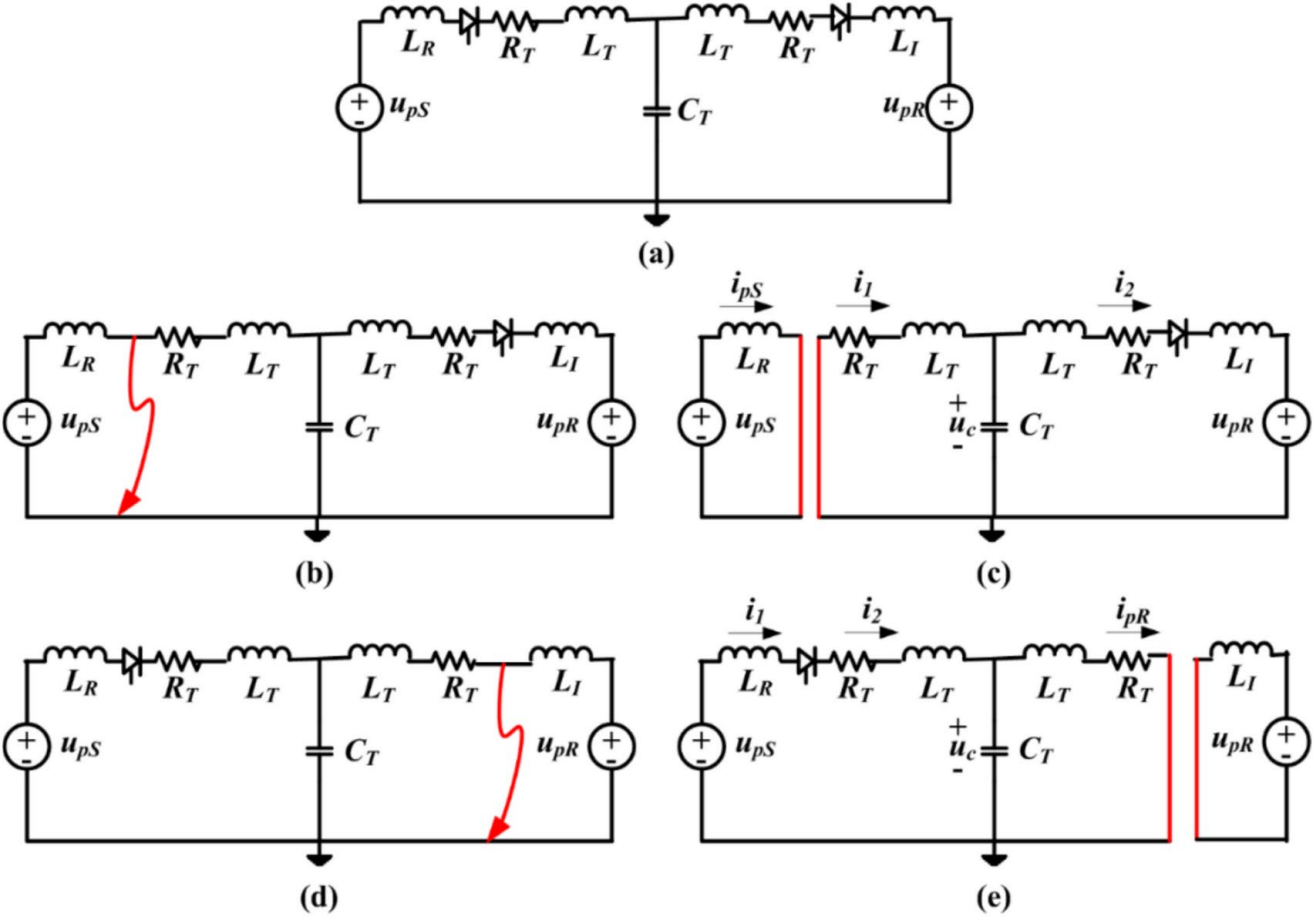
DC line equivalent circuit for (a) Normal operation, (b) Solid fault at rectifier terminal *S*, (c) Equipotential circuit separation for solid fault at *S*, (d) Solid fault at inverter terminal *R,* and (e) Equipotential circuit separation for solid fault at *R*^25^*.*

The equivalent circuit demonstrating the nominal steady state and fault scenarios of the DC line on a per-pole basis is depicted in Fig. 2. The grounding position on the HVDC and nearby AC system influences the impact of P-G-F in the HVDC system. It depends on whether the mid-point of two 12-pulse converters is grounded or the winding of the transformer at high voltage is grounded. The P-G-Fs manifest as either negative pole-to-ground or positive pole-to-ground faults. In Fig. 2, the *R*_*T*_, *L*_*T,*_ and *C*_*T*_ represent the equivalent transmission line parameters for the nominal T model. Similarly, *L*_*R*_ and *L*_*I*_ are the inductances, including smoothing reactor and inductance of HVDC converter at rectifier and inverter, respectively. For a P-G-F incepted during steady-state operation in the positive pole, the *u*_*pS*_ and *u*_*pR*_ are the DC side voltage at the rectifier and inverter terminals. Also, the *i*_*pS*_ and *i*_*pR*_ are the currents at the rectifier and inverter ends^25^.

The post-fault analysis for rectifier and inverter end faults can be governed by the differential expression in (1) and (2), respectively^25^.

1 $$\left\{ \begin{gathered} L_{R} \frac{{di_{pS} }}{dt} = u_{pS} \hfill \\ L_{T} \frac{{di_{1} }}{dt} + i_{1} + u_{c} = 0 \hfill \\ (L_{T} + L_{I} )\frac{{di_{2} }}{dt} + i_{2} R_{T} + u_{pR} = u_{c} \hfill \\ C_{T} \frac{{du_{c} }}{dt} = i_{1} - i_{2} \hfill \\ \end{gathered} \right.$$

2 $$\left\{ \begin{gathered} L_{T} \frac{{di_{2} }}{dt} + i_{2} R_{T} = u_{c} \hfill \\ (L_{T} + L_{R} )\frac{{di_{1} }}{dt} + i_{1} R_{T} + u_{c} = u_{pS} \hfill \\ C_{T} \frac{{du_{c} }}{dt} = i_{1} - i_{2} \hfill \\ \end{gathered} \right.$$

where *u*_*c*_ is the voltage across the shunt capacitor of TL and *i*_*1*_ and *i*_*2*_ are the currents at the rectifier and inverter side of the positive pole, respectively.

## Extension of PDC-based relaying for LCC-HVDC transmission system

The proposed extension of PDC relaying combines two fault detection criteria based on single-end and double-end DC line current measurements to provide fault detection and classification for both bipolar and monopolar operations of LCC-HVDC transmission. The pole differential current-based protection in^26^ detects faults only in bipolar operation and fails to classify the faulty pole. However, it has the advantage of being simple to implement and independent of line charging current, noise interference, and less data processing which are common issues in most differential current, traveling wave-based methods and machine learning techniques. Therefore, an extension of^26^ is suggested to accommodate fault detection in both bipolar and monopolar operations while also enhancing fault classification capability.

### Relaying Principle during bipolar operation by estimating local PDC

The PDC-based protection is a single-ended relaying algorithm that depends on the transient line currents *i*_*pS*_(*t*) and *i*_*nS*_(*t*) measured on the boundary at S of the positive and negative polarity DC line (or poles), respectively. The currents measured on the other boundary R of the positive and negative poles are *i*_*pR*_(*t*) and *i*_*nR*_(*t*), respectively. The sampling frequency used to record the current is 20 kHz.

Similar to^26^, this study proposes constructing a local PDC on the DC line’s end S or R. Initially, the modulus of the measured current at S or R is estimated, followed by taking the time synchronised current difference between *i*_*pS*_(*t*) and *i*_*nS*_(*t*) or *i*_*pR*_(*t*) and *i*_*nR*_(*t*). This time-synchronised current difference at local terminal S or R is referred to as pole differential current (PDC) and is given by (3). Thus, the relaying criterion using PDC-based protection is given below in (4) and (5).

3 $$Local\,PDCs\, = \left\{ \begin{gathered} \Delta i_{S} (t) = (i_{pS} (t) - i_{nS} (t)) \hfill \\ \,\,\,\,\,\,\,\,\,\,\,\,\,\,\,\,\,\,\,\,\,or \hfill \\ \Delta i_{R} (t) = (i_{pR} (t) - i_{nR} (t)) \hfill \\ \end{gathered} \right.$$

4 $$Detection\,of\,fault\,in\,DC\,line\, = \,\,\left\{ \begin{gathered} if\,|\Delta i_{S} (t)|\, \ge I_{th\_b} \, \to \,Fault\,in\,DC\,line \hfill \\ \& \hfill \\ if\,|\Delta i_{S} (t)|\, < I_{th\_b} \, \to \,No\,fault\, \hfill \\ or \hfill \\ if|\Delta i_{R} (t)|\, \ge I_{th\_b} \to Fault\,in\,DC\,line \hfill \\ \& \hfill \\ if\,|\Delta i_{S} (t)|\, < I_{th\_b} \, \to \,No\,fault \hfill \\ \end{gathered} \right.$$

where Δ*i*_*S*_(*t*) and Δ*i*_*R*_(*t*) are the local PDCs when the proposed relaying is implemented, i.e. either at the rectifier end *S* or inverter end R of the DC line, respectively. Also, *I*_*th_b*_ (= 0.25 p.u.) is a predefined threshold setting used to compare PDC during the bipolar operation of the LCC-HVDC transmission system. An internal fault will be detected whenever the criterion (4) is satisfied.

#### Faulty pole selection criterion

The selection of a faulty pole is based on the polarity of the PDC. The proposed scheme initially determines the polarity of the PDC. If the PDC has a positive polarity (i.e. Δ*i*_*b*_ > 0) and also |Δi_b_|≥ *i*_*th_b*_, it indicates an internal P-G-F in the positive pole of the HVDC line. However, if the PDC has a negative polarity (i.e. Δ*i*_*b*_ < 0) but the magnitude |Δ*i*_*b*_|≥ *i*_*th_b*_. In that case, it indicates a P-G-F in the negative pole of the HVDC transmission. Thus, the criterion for selecting the faulty pole can be given as (5).

5 $$Faulty\;pole\;selection = \left\{ \begin{gathered} if \to \Delta i_{S} (t) > 0 \to positive\;pole\;fault \hfill \\ if \to \Delta i_{R} (t) < 0 \to negative\;pole\;fault \hfill \\ \end{gathered} \right.$$

### Relaying principle during monopolar operation by estimating global PDC

**Fig. 3:**
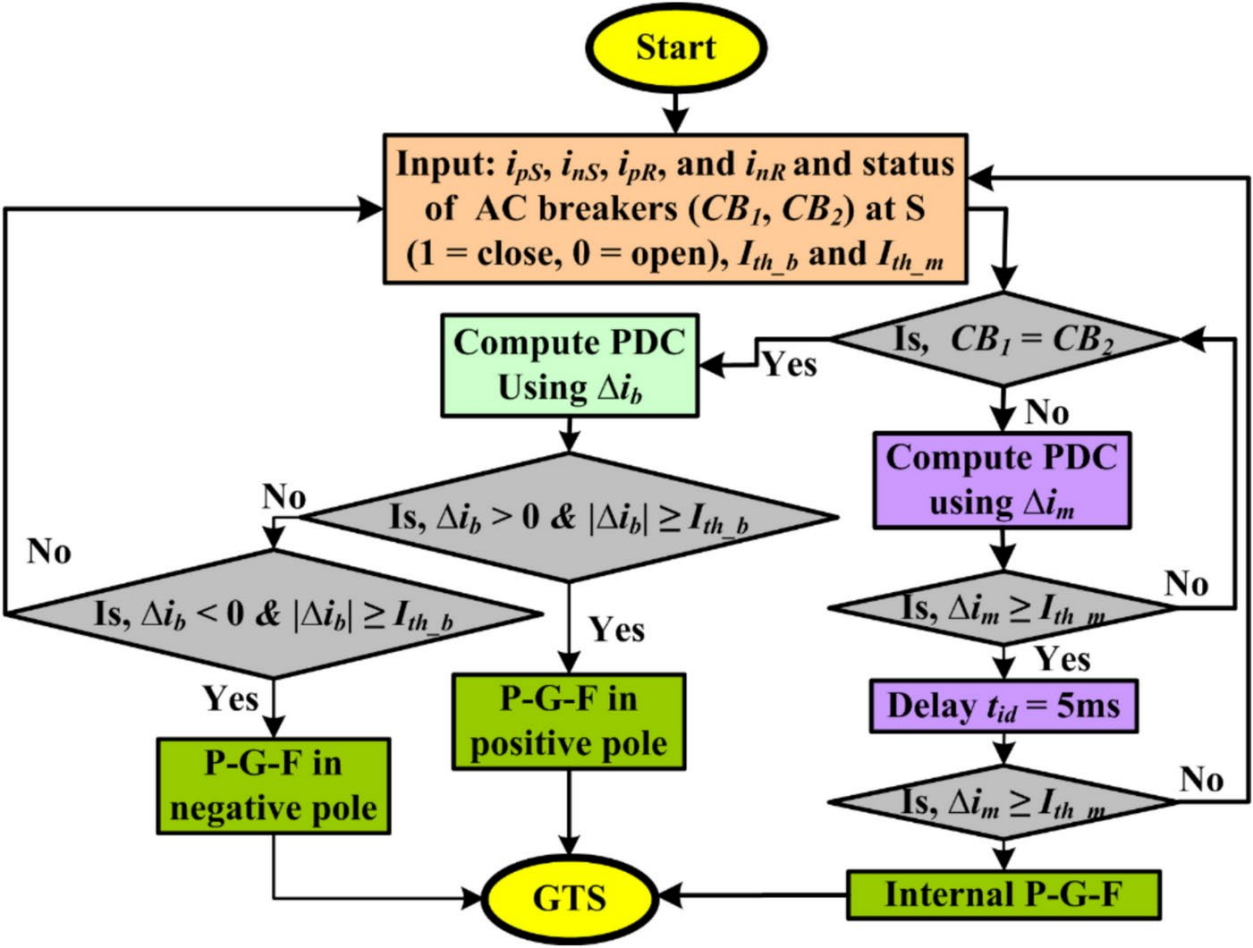
Flowchart for extended PDC-based relaying for HVDC lines.

Through the inaccessibility of one pole due to repairs or permanent faults in the bipolar LCC-HVDC transmission, the operating mode of the HVDC link will be shifted from bipolar to monopolar mode to avoid complete interruption of power flow. Here, the grounded metallic conductor will provide the return path for the DC line current. If the negative pole is out of service for any reason, the estimation of PDC by (3) will be incomplete due to the absence of a negative pole current. Therefore, the algorithm is modified by adding another branch in a flowchart, as shown in Fig. 3. Typically, wireless communication exists between the two ends for data and information exchange. Hence, initially, the status of circuit breakers (*CB*_*1*_, *CB*_*2*_) installed between the feeder and converter transformers at terminal S or R will be monitored to decide the operating mode of the HVDC link. The circuit breaker for the pole under maintenance will be in an open state, while the status of a circuit breaker in the feeder supplying power will be closed. Thus, the proposed extended PDC algorithm decides the path for further processing and selection of the tripping criterion. So, the expression of PDC during monopolar operation (Δ*i*_*m*_) will be given by (6).

6 $$Global\;PDC = \left\{ \begin{gathered} \Delta i_{m} (t) = (i_{pS} (t) - i_{pR} (t)), \to if\;negative\;pole\;is\;out\;of\;service \hfill \\ \quad or \hfill \\ \Delta i_{m} (t) = (i_{nS} (t) - i_{nR} (t)), \to if\;positive\;pole\;is\;out\;of\;service \hfill \\ \end{gathered} \right.$$

where the PDC, Δ*i*_*m*_, is generated using the current difference between two ends of the same pole. This may result in false fault detection due to line capacitance and charging current activation during transients. Therefore, the literature suggests including some intentional time delay (*t*_*id*_) before issuing a trip command by the relaying unit. In^27^, authors proposed a similar communication-based scheme suggesting a time delay *t*_*id*_ of 1.1 s before generating a trip command after fault detection. In^2^, authors suggested keeping a time delay of 100 ms for current differential relaying to avoid maloperation due to line charging current in HVDC lines. Thus, a combination of time delay with a suitable threshold setting (*I*_*th_m*_) during monopolar operation will be preferred for better speed and reliability.

After a numerous simulation study and literature reviews, *I*_*th_m*_ of 0.5 and *t*_*id*_ of 5 ms will provide a trade-off between speed and accuracy during monopole operation^28^. It also avoids malfunctions due to line charging currents during external disturbances. Consequently, if the PDC relaying scheme detects the disturbance at time instant *t*_*d1*_, it takes a delay of *t*_*id*_ to re-verify the disturbance at time instant *t*_*d2*_ (= *t*_*d1*_ + *t*_*id*_). Both the first and second disturbances will be detected if and only if the criterion (7) is satisfied, and then the proposed algorithm will generate a trip command indicating internal P-G-F. However, if the disturbance doesn’t persist for *t*_*id*_ = 5 ms, it would be considered an external disturbance, and the trip command will be restrained. Therefore, an internal fault P-G-F will be detected if (7) is satisfied. However, the faulty pole selection is not required for monopolar operation.

7 $$Detection\;of\;fault\;in\;DC\;line = \left\{ {if\;|\Delta i_{m} (t)| \ge I_{th\_m} \to Fault\;in\;DC\;line} \right.$$

The stepwise explanation and flow chart (Fig. 3) of extended PDC-based relaying suitable for both bipolar and monopolar operation of HVDC link is given as follows:

1. Step 1: Start.
2. Step 2: Declare variable *i*_*pS*_, *i*_*nS*_, *i*_*pR*_, *i*_*nR*_, *CB*_*1*_, *CB*_*2*_, Δ*i*_*b*_ and Δ*i*_*m*_.
3. Step 3: Read the value of *i*_*pS*_, *i*_*nS*_, *i*_*pR*_, *i*_*nR*_, *CB*_*1*_, *CB*_*2*_, I_*th_b*_ = 0.25, I_*th_m*_ = 0.5 and *t*_*id*_ = 5 ms.
4. Step 4: Check breaker positions; if *CB*_*1*_ = *CB*_*2*_ = 1 (both the breakers are closed), consider bipolar mode, calculate Δ*i*_*b*_, and move to step 5. If *CB*_*1*_ ≠ *CB*_*2,*_ i.e., *CB*_*1*_ = 1 and *CB*_*2*_ = 0 (*CB*_*1*_ is closed and *CB*_*2*_ is open) or vice versa, consider monopolar mode, calculate Δ*i*_*m*_, and move to step 6.
5. Step 5: If Δ*i*_*b*_ > 0 & |Δ*i*_*b*_|≥ I_*th_b*_, indicate P-G-F in the positive pole or if Δ*i*_*b*_ < 0 &|Δ*i*_*b*_|≥ I_*th_b,*_ indicate P-G-F in the negative pole, generate a trip signal (GTS); else move to step 3.
6. Step 6: If Δ*i*_*m*_ ≥ I_*th_m*_, add a time delay of *t*_*id*_ = 5 ms and move to step 7. If not satisfying Δ*i*_*m*_ ≥ I_*th_m*_, move to step 4.
7. Step 7: If Δ*i*_*m*_ ≥ I_*th_m*_, generate a trip signal (GTS); else move to step 3.
8. Step 8: End.

### Selection of threshold current (***I***_***th***_)

Ground faults occurring at the farthest location from any terminal of the DC line, with a maximum fault resistance, pose the most significant risk of not being detected by PDC relaying. It may happen due to the excessively low amplitude of PDC for remote-end high-impedance faults. Therefore, protective thresholds should not exceed the minimum value of PDC generated by the highest-impedance remote-end internal faults. Additionally, since the bipolar HVDC line can operate in bipolar or monopolar modes, the selected thresholds should be adjusted accordingly. The separate threshold selection for the two operating modes improves the selectivity and sensitivity of the proposed scheme. From observation, the threshold for the monopolar operating mode is set twice as high as for the bipolar mode to mitigate the effect of distributed line capacitance during transients. This setting will also address the maximum unbalanced current during transients generated due to external faults^23^. Thus, the two sets of thresholds, denoted as (*I*_*th_b*_) and (*I*_*th_m*_), are established for bipolar and monopolar operating modes, respectively.

Thus, the threshold selection is based on extensive simulations to evaluate the proposed scheme under various fault conditions and transition resistances. The transition resistance is varied from 0 to 500 Ω in increments of 50 Ω. At the same time, the fault location is continuously varied from 0 to 900 km, with an intermediate step of 50 km. During the threshold analysis, different types of internal faults, such as pole-to-ground faults (P-G-Fs) and pole-to-pole faults (P-P-Fs), as well as external faults, including line-to-ground faults (L-G-Fs), line-to-line faults (L-L-Fs), and three-phase faults (L-L-Ls), are examined.

After a comprehensive analysis of fault types, locations, and transition resistances, a threshold value of I_th_b_ = 0.25 p.u. is selected, enabling fault detection within less than 15 ms for HVDC transmission systems.

### Time of detection

The typical fault detection time is determined by evaluating the arithmetic mean of the time of detection of fault in the various cases, considering different line length (0–900 km) and fault resistance up to 500 Ω.

## Simulation results and validation

The simulation result and response of extended PDC-based relaying for bipolar and monopolar operating modes of the HVDC link are given in this section. Initially, PSCAD/EMTDC software is used to simulate the test system, and the results are further analyzed using MATLAB/Simulink environment. The reliability and selectivity of the extended PDC relaying are validated by simulating internal faults, external faults and sudden changes in power flow conditions. The internal faults *F*_*1*_ *to* *F*_*4*_ are incepted at distances 0 to 900 km with mutable fault resistance (*R*_*f*_) of 0.1–500 Ω.

### Extended PDC-based relaying response for positive pole faults during bipolar operation

**Fig. 4:**
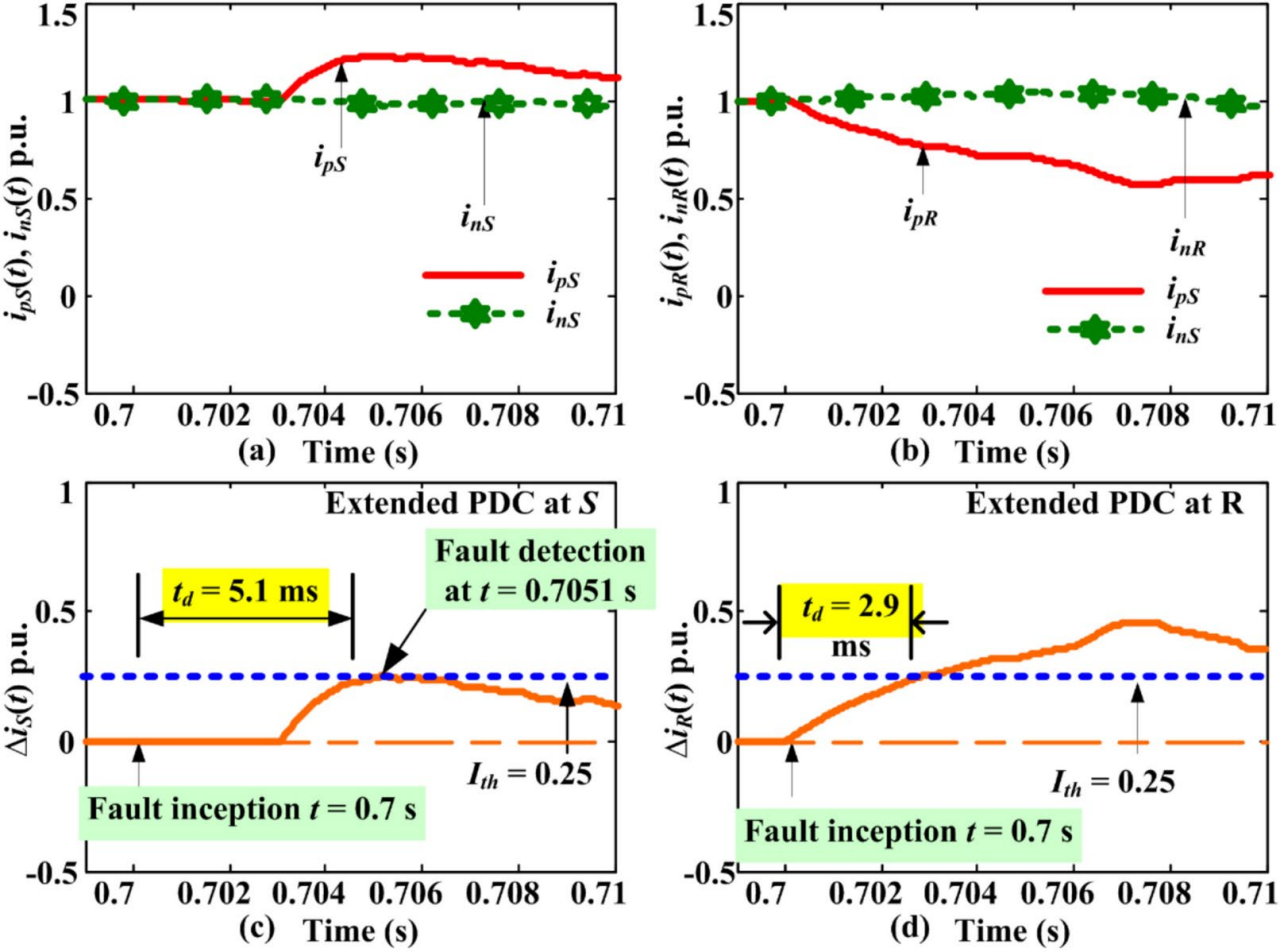
Positive pole DC line currents and PDCs during P-G-F at *F*_*4*_ = 900 km with *R*_*f*_ = 500 Ω.

A pole-to-ground (P-G-F) fault was incepted at 900 km from *S* at t = 0.7 s with a fault resistance of 500 Ω. The results for DC line current in different poles of the HVDC link and PDC estimated using (3) are given in Fig. 4. Results indicated an increase in faulty pole current with a sufficient slope. However, the slope of PDC is higher than that of the pole current. The average slope for pole current is 78.16 p.u. per second, while the average slope for PDC is 82.23 p.u. per second. The PDC crosses the *I*_*th_b*_ after a certain time delay, and the instant of fault detection occurs when the PDC becomes equal to or greater than the *I*_*th_b*_. Figure 4c illustrates the reaction of PDC together with the threshold setting. The relaying unit is at terminal S of the DC line, and *M*_*1*_ and *M*_*3*_ perform the measurements. After fault inception at time *t* = 0.7 s, the increasing trend of PDC rises to 0.26 p.u. with a slope of 17.3 p.u. per second. The time of interception for PDC and *I*_*th*_ is 0.7051 s after fault inception. Thus, the proposed algorithm will generate a trip signal for the control and protection unit after 5.1 ms of the fault inception. Hence, the proposed relaying effectively detects a fault with a high resistance fault of 500 ohms and a location 900 km away from the relaying unit. The maximum fault detection time is 5.1 ms, which is still less than the fault detection time by many current differential algorithms proposed in^23,29–31^.

The fault detection time and status of fault detection measured from line ends S are summarised in Table 1 for faults *F*_*1*_ to *F*_*4*_ with resistance 10–500 ohms. From the results, it can be concluded that for faults close to the line periphery (closer to the measuring unit), i.e., *F*_*1*_ at S, the fault will be noticed within 3.1 ms for an extensive variation of fault resistance up to 500 Ω. Similarly, the maximum fault detection time is 5.1 ms, detected for faults at the far end, 900 km away, with a fault resistance of 500 Ω. Additionally, the average detection time across the entire line length from 0 to 900 km, considering a wide range of fault resistance from 0 to 500 ohms, is 2.87 ms.

**Table 1:** Extended PDC-based relaying response during bipolar operation.

Fault location/type	*R*_*f*_ Ω	Max magnitude of absolute PDC		Fault detection time at *S* and *R* in ms		^#^GTS Yes/No	Classification Faulty pole
		|Δ*i*_*S*_| p.u	|Δ*i*_*R*_| p.u	(*t*_*dS*_)	(*t*_*dR*_)		
*F*_*1*_ = 1 km P-G-F	10	1.40	1.06	0.7	3.4	Internal	Positive
	100	0.94	0.84	0.9	3.6	Yes	Positive
	300	0.53	0.43	1.6	4.0	Yes	Positive
	500	0.31	0.29	3.1	4.8	Yes	Positive
*F*_*2*_ = 300 km P-G-F	10	1.41	1.12	1.4	2.4	Yes	Positive
	100	0.88	0.95	1.7	2.7	Yes	Positive
	300	0.50	0.53	2.5	3.5	Yes	Positive
	500	0.34	0.36	4.7	4.3	Yes	Positive
*F*_*3*_ = 600 km P-G-F	10	1.20	1.13	2.4	1.3	Yes	Positive
	100	0.81	1.09	2.7	1.7	Yes	Positive
	300	0.45	0.61	3.6	2.5	Yes	Positive
	500	0.32	0.42	4.4	4.0	Yes	Positive
*F*_*4*_ = 900 km P-G-F	10	1.13	1.18	3.5	0.7	Yes	Positive
	100	0.70	1.14	3.6	1.0	Yes	Positive
	300	0.35	0.70	4.1	1.8	Yes	Positive
	500	0.27	0.47	5.1	2.9	Yes	Positive
*F*_*5*_ = 600 km P-G-F	500	0.31	0.43	4.4	4.0	Yes	Negative
*F*_*6*_ = 300 km P-P-F	10	0	0	*NA	NA	No	NA
*F*_*S*_—L-G-F	0.01	0	0.1	NA	NA	No	External
*F*_*S*_—L-L-G		0	0	NA	NA	No	External
*F*_*S*_—L-L-L-G		0	0	NA	NA	No	External
*F*_*R*_—L-G-F		0	0	NA	NA	No	External
*F*_*R*_—L-L-G		0	0	NA	NA	No	External
*F*_*R*_—L-L-L-G		0	0	NA	NA	No	External

*NA refers to Not applicable, # refers to Generated trip signal.

where *t*_*dS*_ and *t*_*dR*_ are the times taken to detect faults by PDC relaying implemented at line ends *S* and *R*, respectively.

### Extended PDC-based relaying for negative pole faults

**Fig. 5:**
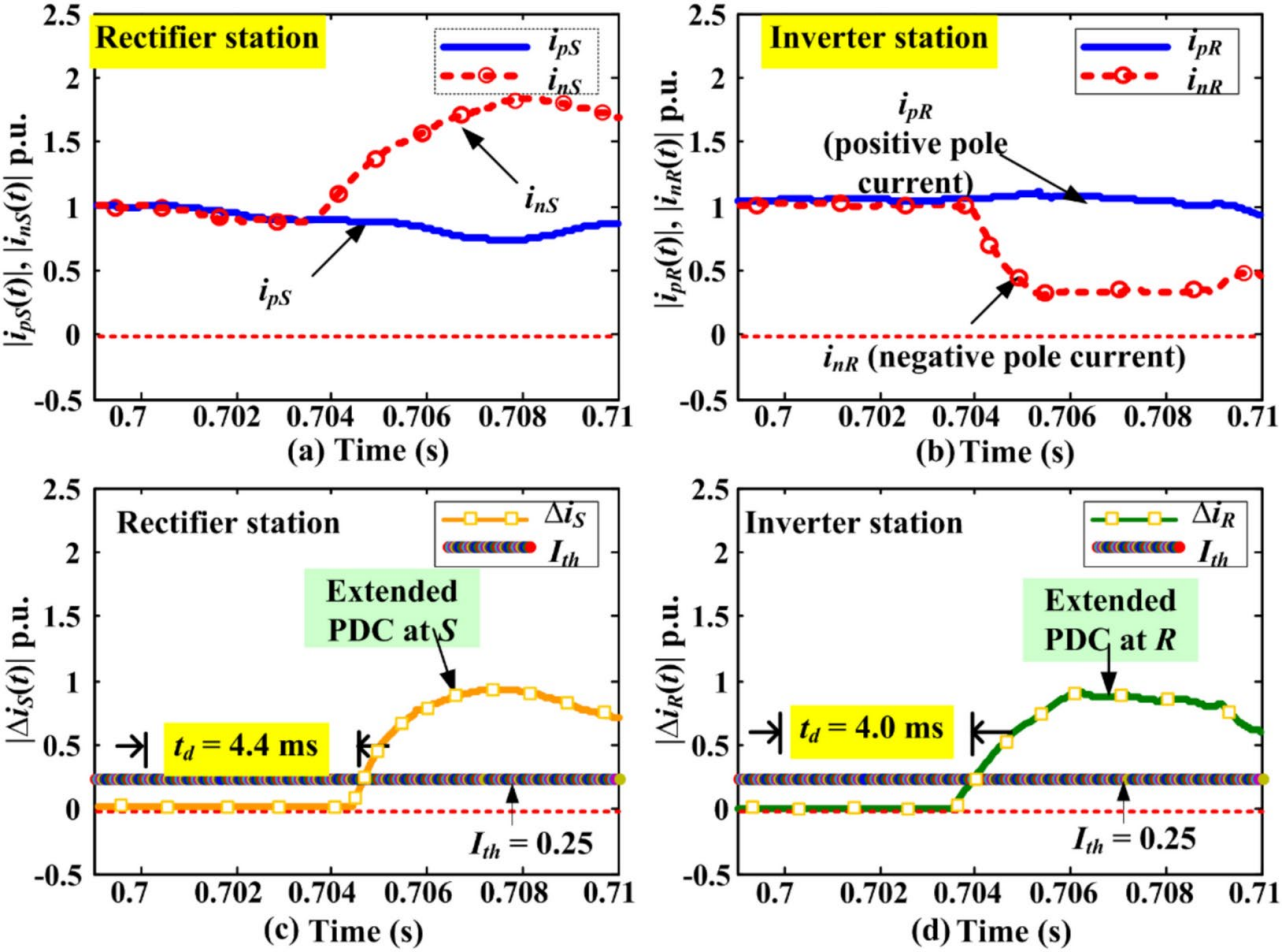
Negative pole DC line current and PDC during P-G-F at *F*_*5*_ = 600 km with *R*_*f*_ = 500 Ω.

The *F*_*5*_ (in Fig. 1) represents a P-G-F incepted on the negative pole of the DC line with fault impedance ranging from 0 to 500 ohms. The fault is initiated at time *t* = 0.7 s and persists for 50 ms. Results indicate that the fault detection time for P-G-F on the negative pole is similar to that for the positive pole under similar fault circumstances. Therefore, the study for *F*_*5*_ at 600 km and *R*_*f*_ of 500 Ω is illustrated in Fig. 5. Here, the fault detection time by PDC at S and R is 4.4 ms and 4.0 ms, respectively. The maximum amplitude of the negative pole current at measuring unit *M*_*3*_ is 1.31 p.u., and at *M*_*4*_ is 0.43 p.u., whereas the current for the positive pole shows minimal change after fault inception.

### Extended PDC-based relaying for pole-to-pole faults (P-P-Fs)

**Fig. 6:**
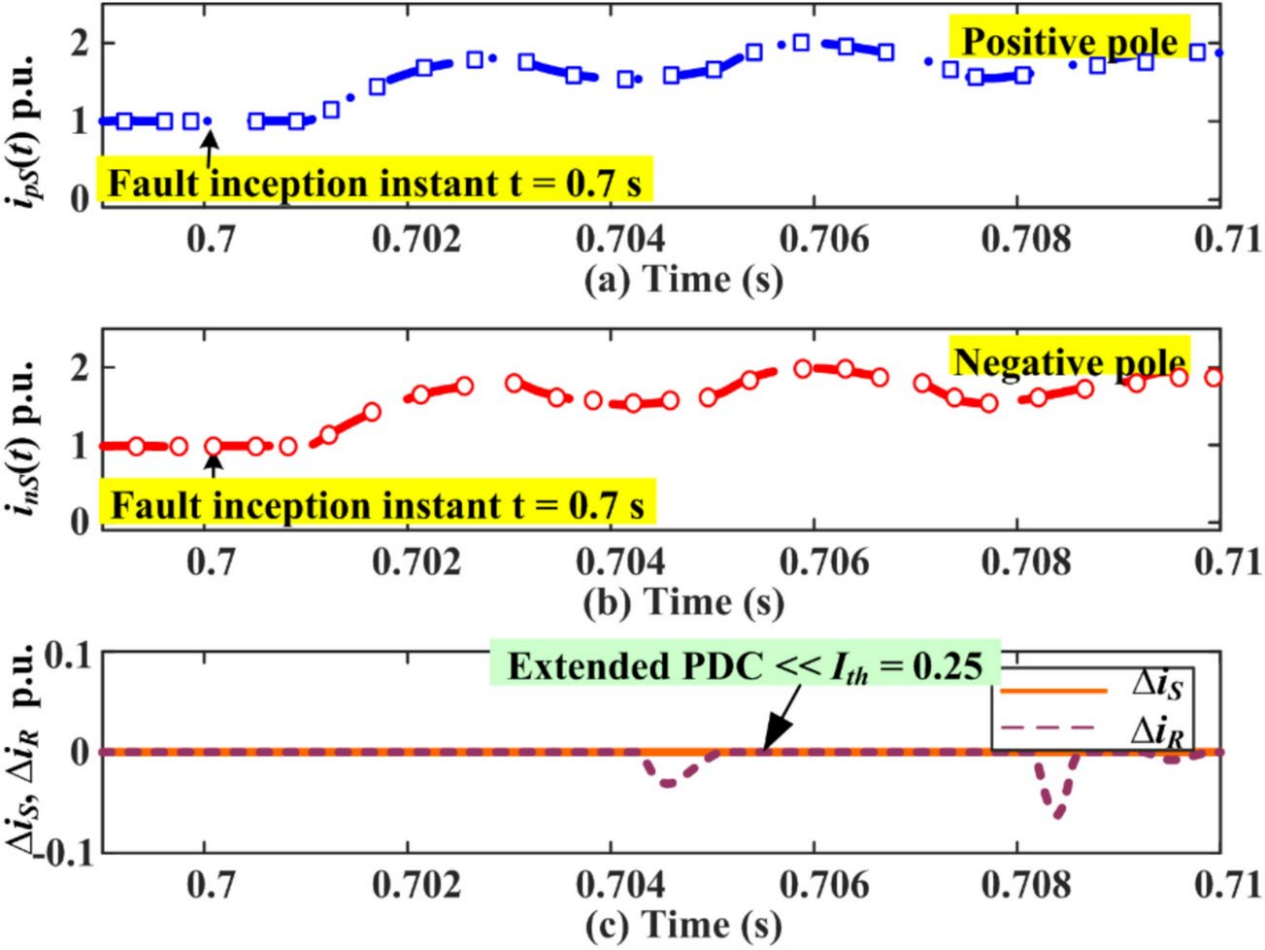
Extended PDC-based relaying response for P-P-F at 300 km and *R*_*f*_ = 10 Ω.

A double pole fault (P-P-F) is simulated on the HVDC test system (as depicted by *F*_*6*_ in Fig. 1) at 300 km from terminal *S* with a fault resistance of 10 Ω. The rise in pole current and PDC for the two poles of the HVDC transmission is illustrated in Fig. 6. The results demonstrate that the maximum increase in pole current is 1.7 p.u. for both positive and negative poles of HVDC lines. However, the PDC obtained from the relaying unit at terminal S is absolutely zero, while the PDC obtained from the relaying unit at *R* has a maximum amplitude of 0.04 p.u. at 0.45 ms. The PDC at either S or R relaying locations is less than the predefined threshold setting. Consequently, the relaying criterion of fault detection remains unsatisfied, and the algorithm will not issue any trip command. Hence, the relaying criterion proposed (4) cannot detect any P-P-F incepted on the HVDC system.

### Extended PDC-based relaying for external AC-side faults

**Fig. 7:**
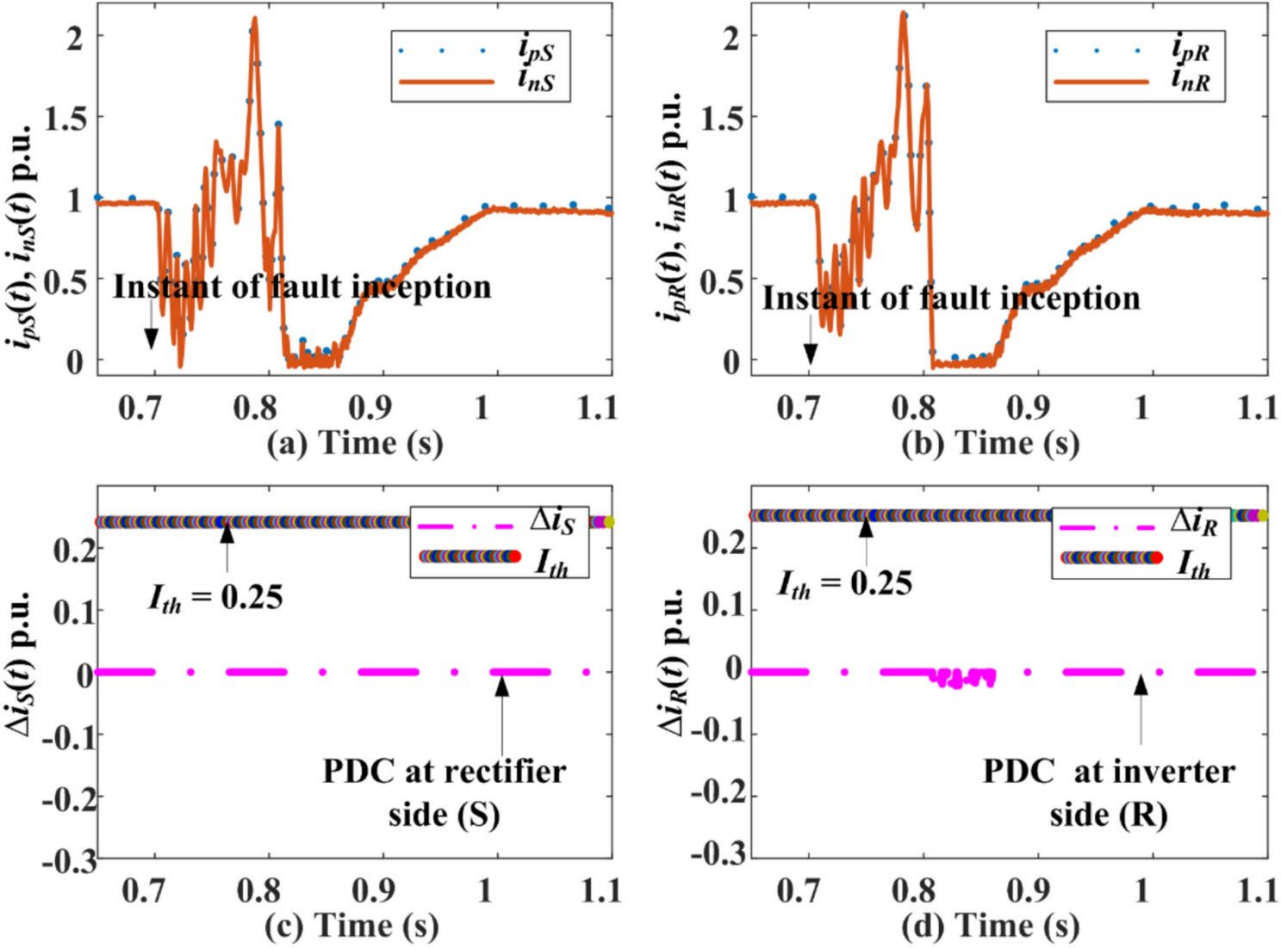
PDC-based relaying response during AC-side L-G-F at *F*_*S*_*.*

**Fig. 8:**
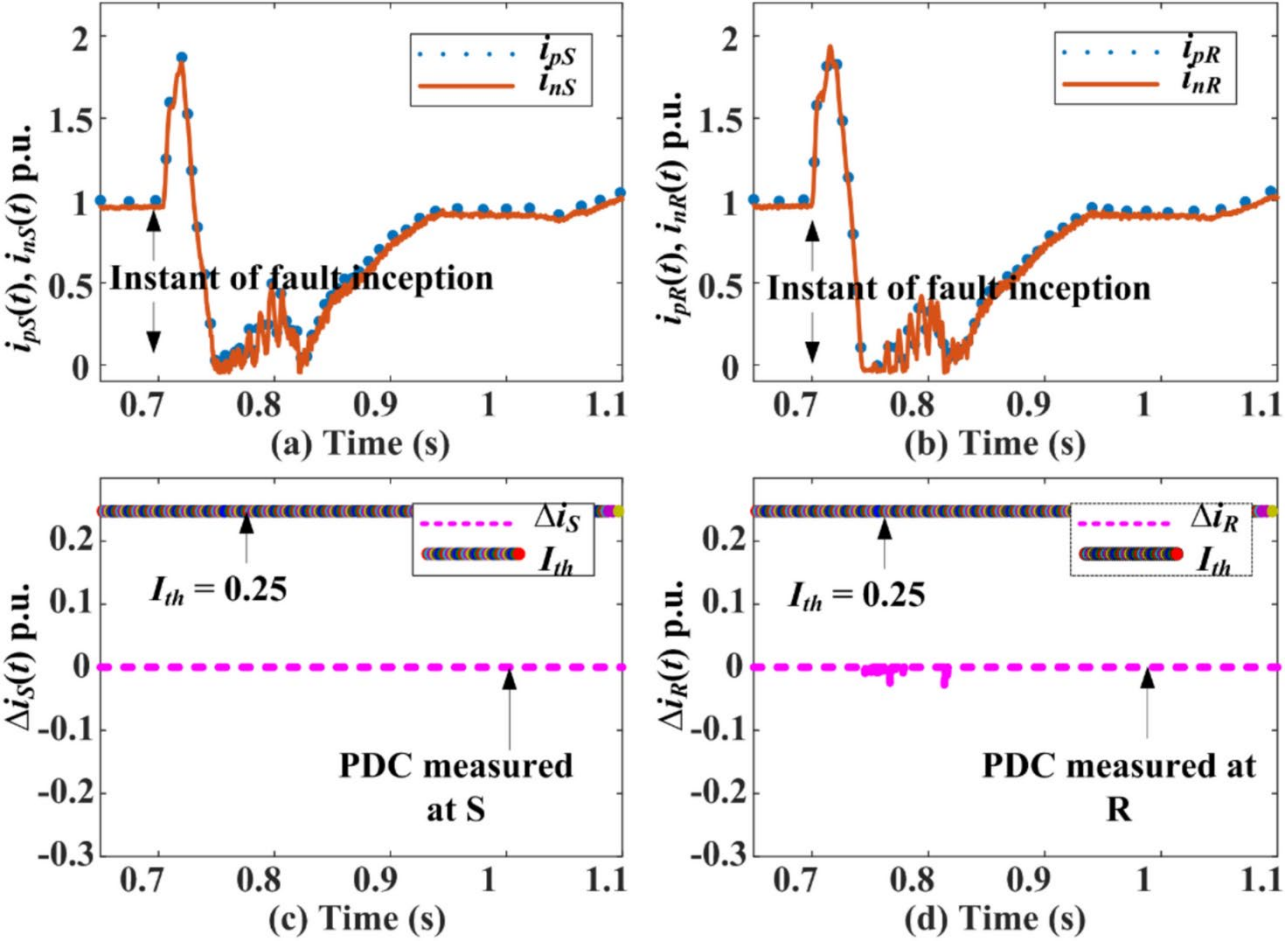
PDC response during AC-side L-G-F at *F*_*R*_*.*

Three types of external shunt faults are simulated: line-to-ground fault (L-G-F), line-to-line-to-ground (L-L-G), and triple line-to-ground (L-L-L-G) at *F*_*S*_ and *F*_*R*_ in the HVDC test system shown in 1. The response for the pole currents, the maximum magnitude of PDC, fault detection time from both relaying units present at *S* and *R*, and the status of the trip signal (GTS) during faults at *F*_*S*_ and *F*_*R*_ are indicated in Fig. 7 and Fig. 8. High transient appears in the current of each pole of DC line after fault inception as illustrated in Fig. 7 for an L-G-F at location *F*_*S*_. The PDC measured at terminal *S* is unaffected, while the PDC at terminal *R* is affected minimally. However, the PDCs calculated at terminal *S* or *R* are less than the pre-selected threshold setting of 0.25 p.u. Therefore, the extended PDC-based relaying unit is entirely stable for external L-G-F at *F*_*s*_. The results for L-L-G and L-L-L-G faults at *S* are illustrated and highlighted in Table 1. Similarly, Fig. 8 is concerned with an L-G-F at fault location *F*_*R*_, showing a quick rise in the current of each pole after fault inception. The amplitude of the increase in current in both poles is nearly similar to 2 p.u. Hence, the magnitude of PDC calculated from the relaying unit installed at terminal *S* is zero, while the PDC estimated at terminal *R* is 0.05 p.u. The resultant PDC is less than the threshold setting of 0.25 p.u in both conditions. Thus, the scheme is stable for external faults on both the rectifier and inverter side of the HVDC system. The results indicate that the extension of PDC relaying is stable for each external fault and does not generate any tripping signal during transients generated by external faults.

### Simulation results for Extended PDC-based relaying during Monopolar Operation

**Fig. 9:**
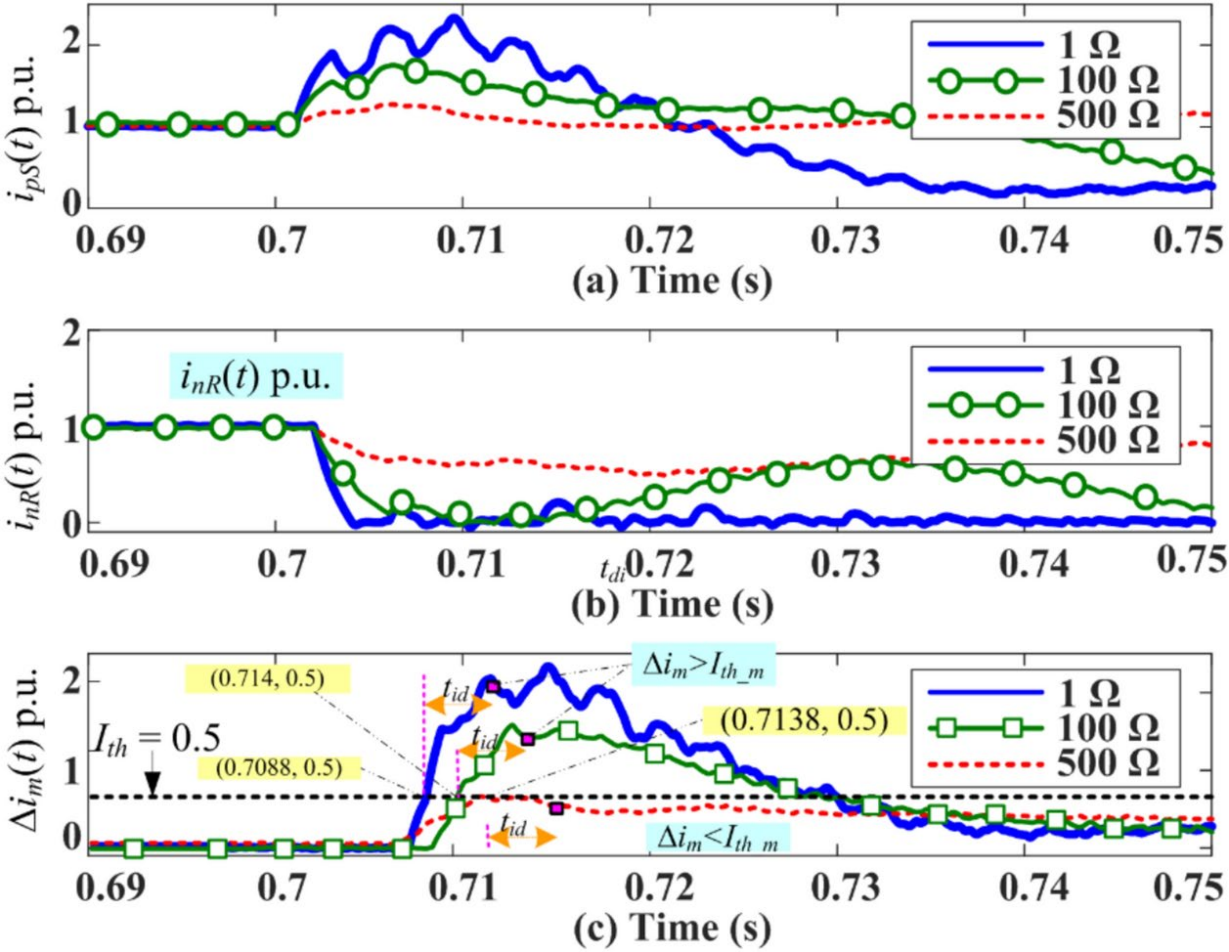
Pole current and PDC during monopolar operation for P-G-F at *F*_*2*_ = 300 km from terminal *S.*

This section examines the response for the extended PDC-based algorithm during monopolar operation. Internal P-G-Fs *F*_*1*_ to *F*_*4*_ are simulated with variable fault resistances of 1 Ω, 100 Ω, 300 Ω, and 500 Ω at each location. The results of an illustrative case of fault at location *F*_*2*_ are depicted in Fig. 9, while the remaining results are tabulated in Table 2. The results in Fig. 9 show the pole currents at metering points *M*_*1*_ (i.e. *i*_*pS*_), *M*_*2*_ (i.e. *i*_*pR*_), and PDC (Δ*i*_*m*_) for the HVDC pole in service, i.e. positive pole. Table 2 illustrates the maximum amplitude of currents measured at two ends of TL, the maximum amplitude of PDC, the time of first detection (*t*_*d1*_), and the second detection time (*t*_*d2*_). The *t*_*d1*_ represents the instant when PDC exceeds the threshold *I*_*th_m,*_ including a communication delay of 7 ms^28^ involved during PDC calculation. Similarly, the second detection time, *t*_*d2*_, occurs after an intentional time delay (*t*_*id*_) of 5 ms (as given in the proposed algorithm flowchart Fig. 3). The proposed relaying scheme generates a trip signal only if the relaying criterion is satisfied at *t*_*d2*_, and else it is restrained.

**Table 2:** Extended PDC-based relaying response during monopolar operation.

Fault location/type	*R*_*f*_ Ω	|*i*_*pS*_|_max_ p.u	|*i*_*pR*_|_max_ p.u	|Δ*i*_*m*_|_max_ p.u	*t*_*d1*_ (ms)	*t*_*id*_ (ms)	*t*_*d2*_ (ms)	Generate trip signal (GTS)
F_1_ = 1 km P-G-F	1	2.24	0.01	2.21	8.3	5	13.3	Yes
	100	1.74	0.13	1.61	9.1	5	14.1	Yes
	300	1.4	0.40	1.0	9.8	5	14.8	Yes
	500	1.3	0.70	0.62	12.9	5	NA	No
F_2_ = 300 km P-G-F	1	2.32	0.02	2.3	8.8	5	13.8	Yes
	100	1.69	0.11	1.58	9.4	5	14.4	Yes
	500	1.27	0.66	0.60	12.2	5	NA	No
F_3_ = 600 km P-G-F	1	2.02	0.01	2.01	8.8	5	13.8	Yes
	100	1.6	0.0	1.60	9.3	5	14.3	Yes
	300	1.38	0.45	0.93	10.4	5	15.4	Yes
	500	1.24	0.65	0.59	12.1	5	NA	No
F_4_ = 900 km P-G-F	1	2.08	0.0	2.09	8.4	5	13.4	Yes
	100	1.57	-0.01	1.58	9.4	5	14.4	Yes
	300	1.36	0.50	0.92	10.6	5	15.6	Yes
	500	1.2	0.70	0.40	12.2	5	NA	No
F_S_—L-G-F	0.01	1.03	1.53	0.57	12.2	5	NA	No
F_S_—L-L-G		0.13	0.9	0.73	9.7	5	NA	No
F_S_—L-L-L-G		0.13	0.97	0.77	8.6	5	NA	No
F_R_—L-G-F		1.9	2.08	0.48	-	5	NA	No
F_R_ L-L-G		2.12	1.834	0.72	8.9	5	NA	No
F_R_—L-L-L-G		0.72	0.71	0.73	8.7	5	NA	No

Extensive simulation tests have been conducted for internal faults (1–900 km) and external faults *F*_*S*_ and *F*_*R*_ with variable fault resistances, as shown in Table 2. The results indicate that the scheme successfully distinguishes the internal and external faults under each condition but with fault resistance (*R*_*f*_) up to 300 ohms. For *R*_*f*_ beyond 300 Ω, it is observed that the scheme fails to detect internal faults. It can be observed in the PDC response in Fig. 9c for fault resistance 500 ohms, where the relaying criterion is satisfied at *t*_*d1*_ (i.e. Δ*i*_*m*_ ≥ *I*_*th_m*_), while at *t*_*d2*_, the same criterion is not satisfied (i.e. Δ*i*_*m*_ < *I*_*th_m*_).

### Extended PDC-based relaying response for cross-country and evolving faults

A cross-country fault is characterized by an earth fault occurring in various phases of the same circuit but at different locations and times. Additionally, evolving faults start in one phase and extend to another phase after several cycles^32^ of AC supply. However, these faults are considered as follows for HVDC transmission: A cross-country fault is a ground fault initiated at the AC side, leading to another ground fault at the DC side of HVDC transmission. For example, an L-G-F on the inverter-side AC lines close to the converter station may cause another P-G-F on the DC line due to excessive momentary current. Similarly, evolving faults are considered a P-G-F in one pole of a bipolar HVDC line, followed by a P-G-F in another pole of the HVDC system. Timely detection of these faults is challenging because the former involves faults occurring at distinct locations. At the same time, the latter entails a transition in fault type after a few seconds.

**Fig. 10:**
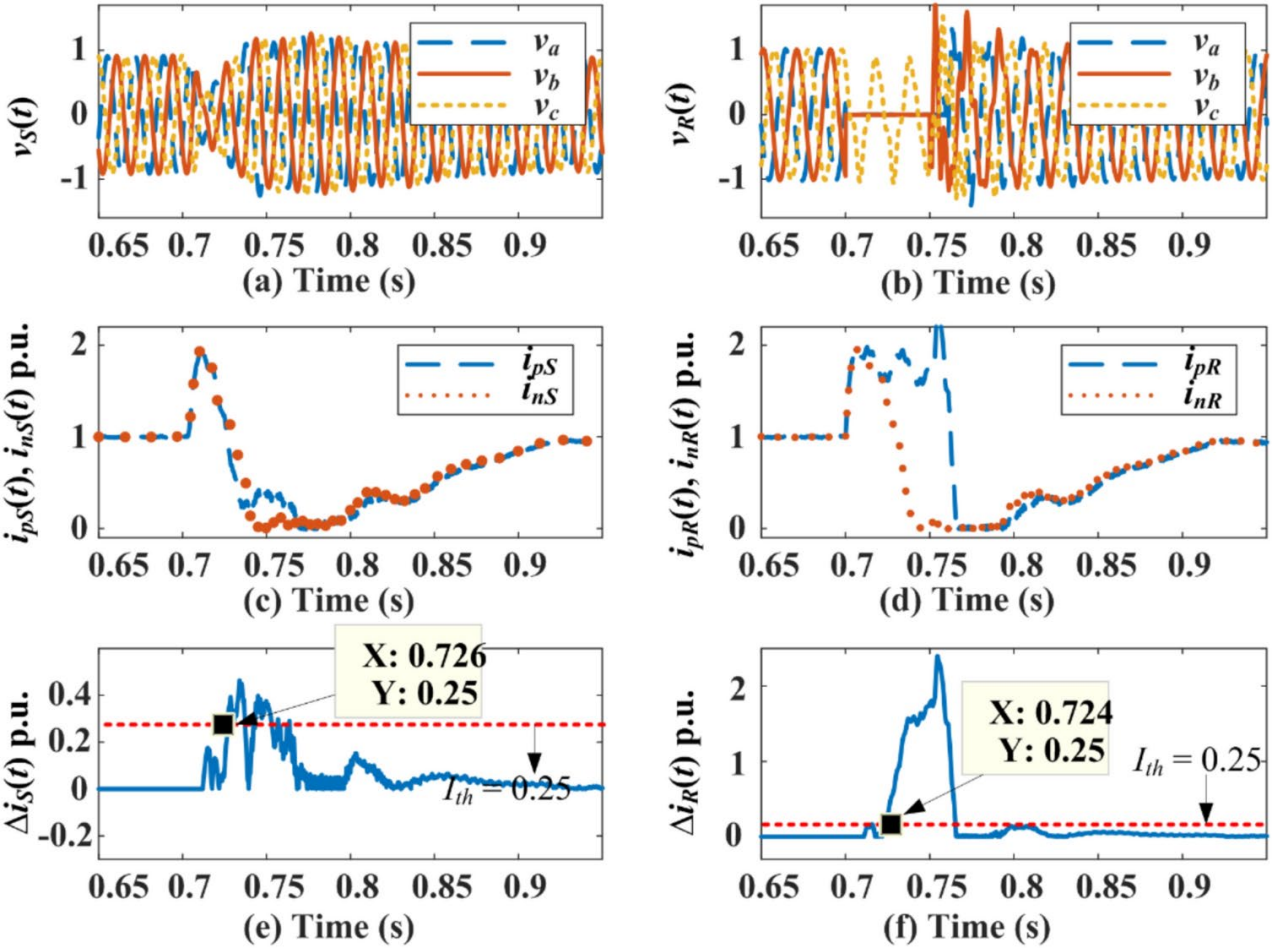
Cross-country fault detection for faults incepted at *F*_*R*_ and *F*_*3*_*.*

**Fig. 11:**
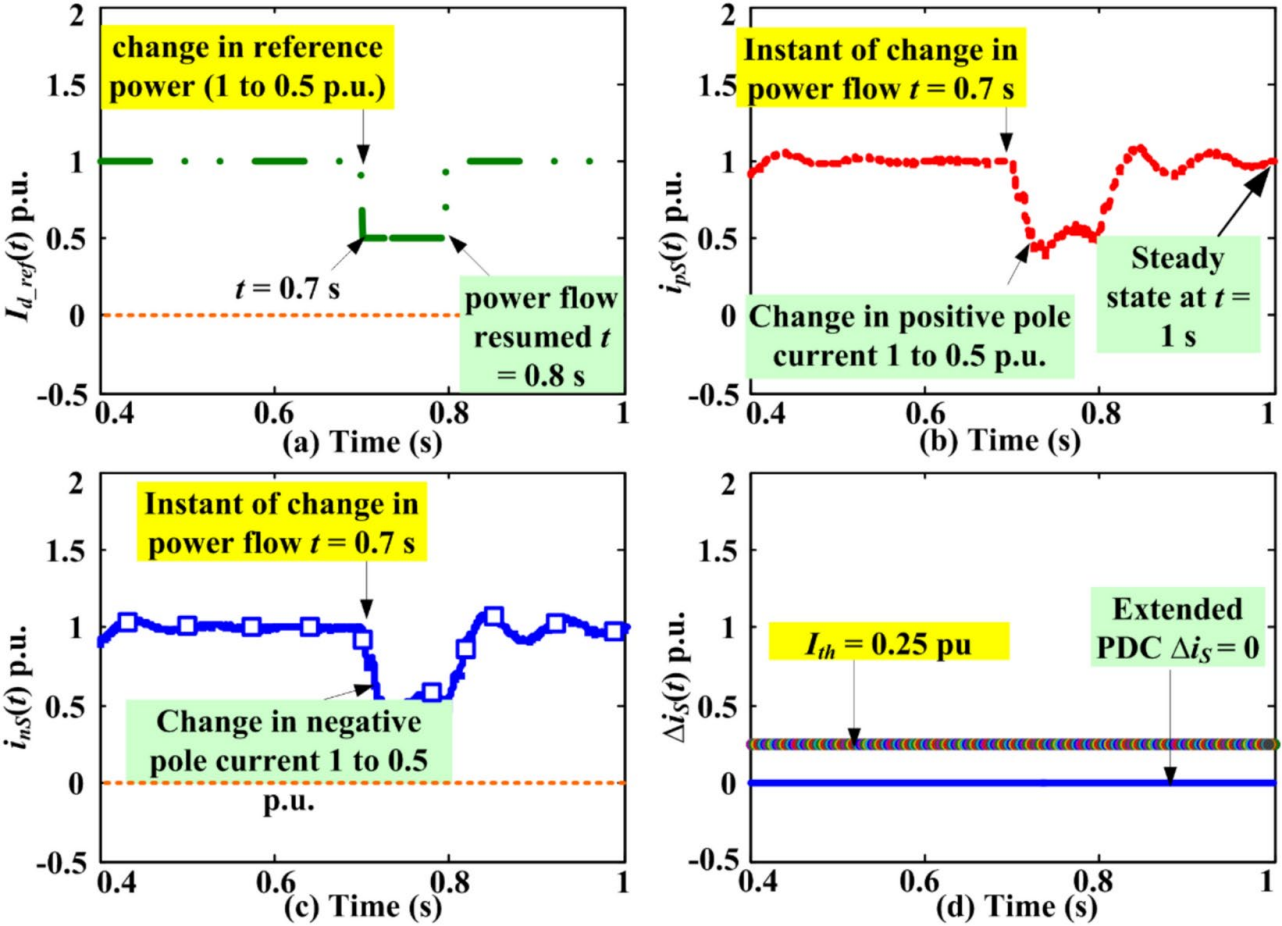
DC line current and PDC response due to abrupt change in power flow.

This section investigates the PDC response during cross-country and evolving faults. The results for one case are illustrated in Fig. 11, while the others are presented in Table 3. In Fig. 10, an L-L-G fault was initiated at time *t* = 0.7 s and cleared at time *t* = 0.75 s at location *F*_*R*_, leading to another P-G-F fault after 10 ms initiated on the positive pole at 600 km (*F*_*3*_) from *S*. Initially, both pole currents, *i*_*pS*_, *i*_*pR*_, and *i*_*nS*_, *i*_*nR*_, were precisely similar. Still, after 10 ms, *i*_*pS*_ and *i*_*pR*_ displayed opposite variations due to another P-G-F fault on the positive pole. The subsequent P-G-F fault is initiated at t = 0.71 s and is detected after 16 ms and 14 ms by the PDC-relaying units at terminals *S* and *R*, respectively.

**Table 3:** Status of cross-country and evolving fault detection by PDC relaying.

Initiating fault	Subsequent fault	Change in PDC	Fault type	Occurrence possibility	GTS
*F*_*1*_	*F*_*2*_, *F*_*3*_, or *F*_*4*_	No	Cross-country	Rare	No
*F*_*2*_	*F*_*5*_	No	Evolving	Likely	No
*F*_*S*_	*F*_*2*_	No	Cross-country	Rare	No
*F*_*R*_	*F*_*3*_	Yes	Cross-country	Most	Yes

### Extended PDC-based relaying response for abrupt change in power flow

Another scenario involving sudden fluctuations in reference power transmitted via an HVDC link is being examined to assess how PDC relaying responds to these modifications in power transferring over the HVDC lines. Firstly, the power (*P*_*d*_) transmitted over HVDC lines are maintained at 1 p.u., but afterwards, it is minimised to 0.5 p.u. at a specific moment during the simulation. This 50% reduction in power flow is obtained by adjusting the current reference at 0.7 s after starting the simulation for the typical LCC-HVDC model given in Table A1 of the Appendix (or shown in Fig. 1). This dynamic scenario may occur in master control centres due to alterations in the topology of the AC network present connected to the HVDC transmission. The power adjustment persists for a duration of 100 ms, and at the time moment of 0.8 s, the original power supply from the DC line is re-established. Consequently, the varied pole currents and PDC responses are assessed and compared against the threshold. Figure 11 illustrates the dynamics of current in the positive polarity DC line and negative polarity DC line with PDC implemented for the detecting faults scheme at terminal S.

Since the master-control is uniform for positive polarity and negative polarity DC lines, there will be an analogous fluctuation witnessed for the current in both. The poles of positive and negative polarities together adapt such dynamics produced by the master control station. The comparative graph of PDC and *I*_*th*_ in Fig. 11d suggests that the PDC unswervingly preserves a zero magnitude throughout the system dynamics, i.e., it is always less than *I*_*th*_. Therefore, PDC relaying will not generate any trip signals and will remain stable during the dynamic operation of the HVDC system.

### Comparison and practical feasibility of the proposed scheme

A quantitative comparison of the proposed scheme with other recent schemes available in the literature is summarized in Table 4. Table 4 illustrates a direct comparison between the proposed scheme and other schemes available in references^20,26,33–37^. This comparison is based on various factor such as sampling frequency *f*_*s*_, measuring parameter (MPs), *R*_*f*_, trip time, delay in signal processing (DSP), operating modes, protection features, accuracy in time synchronization and noise signal susceptibility (NSS). The validation of proposed scheme stability during cross-country and evolving faults (CCE) is also investigated. It indicates that the proposed relaying is comparatively better due to lower *f*_*s*_, only one MPs, better protection features, trip time, ATS and better NSS. Also, the results of CCE fault validation show that it is stable during operation.

**Table 4:** Comparison of the proposed scheme with the schemes available in the literature.

Features	Proposed scheme	Relaying schemes for bipolar LCC-HVDC system						
		^26^	^33^	^34^	^35^	^36^	^37^	^20^
*f*_*s*_ (kHz)	20	20	10	4.8	200	20	10	10
MPs	*i*,	*i*	*i*, *v*	(*i*_*pS*_–*i*_*pR*_)	*v*	*i*, *v*	*i*, *v*	*i*, *v*
*R*_*f*_ (Ω)	500	500	500	500	300	1000	150	200
Trip time (ms)	5.1 or 15^€^	5.1	2.0	14	4.4	10	6	40
DSP (ms)	7^€^	–	13.3	7.09	–	–	–	–
Operating modes	BO, MO	BO	BO, MO	BO, MO	BO	–	BO, MO	BO, MO
Protection features	FD, FS	FD	FD	FD	FD	FD	FD, FC	DF
ATS	NR	NR	High	High	High	High	–	NR
NSS	High	High	Less	High	Less	Less	–	Less
CCE stability	Yes	NA	NA	NA	NA	NA	NA	NA

€ = MO, FD = fault detection, FS = faulty pole selection, *v* = voltage, *i* = current, BO = bipolar operation, MO = monopolar operation, MP = measuring parameter, ATS = accuracy in time synchronization, NR = not required, NSS = noise signal susceptibility, CCE = cross-country and evolving faults, NA = not available.

The proposed scheme requires only the line current measurement at a single terminal for bipolar operation and at both terminals for monopolar operation of the HVDC line. Additionally, the relatively low sampling rate and minimal number of required measuring parameters enhance the practicality of implementing this scheme. These factors make it a cost-effective and feasible solution for real-world HVDC protection systems.

### Practical implementation feasibility

The proposed scheme requires only the line current measurement at a single terminal for bipolar operation and both terminals for monopolar operation of the HVDC line. Additionally, the relatively low sampling rate and minimal number of required measuring parameters enhance the practicality of implementing this scheme. These factors make it a cost-effective and feasible solution for real-world HVDC protection systems.

## Conclusion

This paper presents an extended single and double-ended measurement-based relaying for LCC-HVDC transmission lines, utilising the transient current measurement at the boundary of positive and negative polarity transmission lines. Combining single and double-ended measurements aims to develop a reliable protection criterion for both bipolar and monopolar modes of operation of the LCC-HVDC transmission system. The transient current at the line boundary defines local and global PDCs to develop the protection criteria. A fault detection criterion is formulated based on the amplitude of PDCs, and the selection of a faulty line (pole) criterion is based on the polarity of PDCs. The key findings of this study are as follows:

1. Initially, based on the status of circuit breakers near converter transformers, the algorithm decides the operating mode of the LCC-HVDC system.
2. In the case of the bipolar mode of operation of the LCC-HVDC transmission, the local PDC appearance with rising amplitude is compared with the threshold to initiate the trip signal. Conversely, during an external fault, the local PDC remains zero.
3. In the case of monopolar operation, the global PDC becomes non-zero during internal disturbances. However, after internal fault inception it crosses the threshold setting and initiates the trip signal to acknowledge fault detection, due to single pole operation faulty pole selection is not required.
4. The proposed method does not rely on a communication channel during bipolar operation. Hence, fault detection is fast, within 5.1 ms for transition resistance up to 500 Ω and fault location at 900 km. The faulty pole is classified based on local PDC polarity. However, during monopolar operation, the communication channel is involved in global PDC calculation. Thus, the speed of fault detection reduces slightly, and the detection for the highest transition resistance up to 300 Ω at fault location 900 km is completed within 15.6 ms.
5. Cross-country and evolving fault conditions are also analyzed through numerous simulations, demonstrating the satisfactory performance of the proposed scheme.

## Supplementary Information

Supplementary Information.

## Data Availability

All the data generated or analysed during this study are included in this article.

## Abbreviations

ATS: Accuracy in time synchronization
ANN: Artificial neural network
CCE: Cross-country and evolving faults
CB: Circuit breaker
DMR: Dedicated metallic return
HVDC: High voltage direct current
GTS: Generate trip signal
LCC: Line commutated converter
L-L, LLL: Line-to-line, three-phase fault
ML: Machine learning
NSS: Noise signal susceptibility
PDC: Pole differential current
P-G-F: Pole-to-ground fault
P-P-F: Pole-to-pole fault
P.U.: Per-unit
TWs: Traveling waves

## List of symbols

Δ*i*_*S*_: PDC at line boundary ‘*S*’ (p.u.)
Δ*i*_*R*_: PDC at line boundary ‘*R*’ (p.u.)
Δ*i*_*m*_: PDC for monopolar operation (p.u.)
*C*_*T*_: Equivalent line capacitance in (Farad)
*f*_*s*_: Sampling frequency (Hz)
*i*_*pS*_, *i*_*pR*_: Positive pole current at end ‘*S*’ and ‘*R*’
*i*_*nS*_, *i*_*nR*_: Negative pole current at end ‘*S*’ and ‘*R*’
*I*_*th_b*_: Threshold for bipolar operation (p.u.)
*I*_*th_m*_: Threshold for monopolar operation (p.u.)
*R*_*f*_: Transition resistance in Ω
*R*_*T*_,: Transmission line resistance in Ω
*P*_*dc*_: LCC-HVDC system Rated power (MW)
*t*_*id*_: Intentional time delay (ms)
*t*_*dS,*_*t*_*dR*_: Fault detection time (ms)
*u*_*c*_: Line shunt capacitances voltage (p.u.)
*u*_*pS*_, *u*_*pR*_: Pole voltage at end ‘S’ and ‘R’ (p.u.)
