# Supplementary material for: Extension of pole differential current based relaying for bipolar LCC HVDC lines
Source: Sci Rep. 2025 May 9;15:16142. doi: 10.1038/s41598-025-94842-0 (PMC12062258; doi:10.1038/s41598-025-94842-0)
Supplement: Supplementary file 1 — Supplementary Information. [file 41598_2025_94842_MOESM1_ESM.docx]

**SUPPLEMENTARY MATERIAL**

**APPENDIX A**

**TABLE A1.** System parameters @ LCC-HVDC transmission

| **Parameters** | **Rectifier station** | **Inverter station** |
| --- | --- | --- |
| HVDC transmission type | Bipolar and Monopolar | |
| Transmission capacity (*P_dc_*) in MW | 1000 MW per pole | |
| Voltage capacity (*U_d_*) kV | ± 500 kV for each pole | |
| Current capacity (*I_d_*) kA | 2 kA per-pole | |
| Converters classification | LCC | LCC |
| Converter capacity | 1200 MVA per pole | 1200 MVA per pole |
| Grid capacity | 500 kV, 60 Hz, 5000 MVA, | 345 kV, 50 Hz, 10,000 MVA |
| Converter transformers at | 1200 MVA, 60 Hz | 1200 MVA, 50 Hz |
| Smoothing Reactor (*L_s_*) | 0.5 H on the boundaries of each pole | 0.5 H on the boundaries of each pole |
| Capacity of AC Filters at | 1200 Mvar, 60 Hz | 1200 Mvar, 50 Hz |
| Minimum Angle | α = 13^0^ | γ = 18^0^ |
| SCR | 2.5 | 5 |

**TABLE A2.** Simulated distributed transmission line parameter

| **Parameter** | **Symbol and unit** | **Rating** |
| --- | --- | --- |
| Resistance | *R* Ω/km | 0.0267 |
| Inductance | *L* H/km | 0.791e-3 |
| Capacitance | *C* F/m | 14.3e-9 |
| Length of transmission | *L_t_* km | 900 km |
